# Tracking SARS-CoV-2 variants of concern in wastewater: an assessment of nine computational tools using simulated genomic data

**DOI:** 10.1099/mgen.0.001249

**Published:** 2024-05-24

**Authors:** Steven G. Sutcliffe, Susanne A. Kraemer, Isaac Ellmen, Jennifer J. Knapp, Alyssa K. Overton, Delaney Nash, Jozef I. Nissimov, Trevor C. Charles, David Dreifuss, Ivan Topolsky, Pelin I. Baykal, Lara Fuhrmann, Kim P. Jablonski, Niko Beerenwinkel, Joshua I. Levy, Abayomi S. Olabode, Devan G. Becker, Gopi Gugan, Erin Brintnell, Art F.Y. Poon, Renan Valieris, Rodrigo D. Drummond, Alexandre Defelicibus, Emmanuel Dias-Neto, Rafael A. Rosales, Israel Tojal da Silva, Aspasia Orfanou, Fotis Psomopoulos, Nikolaos Pechlivanis, Lenore Pipes, Zihao Chen, Jasmijn A. Baaijens, Michael Baym, B. Jesse Shapiro

**Affiliations:** 1 Department of Microbiology and Immunology, McGill University, Montreal, QC, Canada; 2 Environment and Climate Change Canada, Montreal, QC, Canada; 3 Department of Biology, University of Waterloo, Waterloo, ON, Canada; 4 Department of Biosystems Science and Engineering, ETH Zurich, Basel, BS, Switzerland; SIB Swiss Institute of Bioinformatics, Lausanne, VD, Switzerland; 5 Department of Immunology and Microbiology, The Scripps Research Institute, La Jolla, CA, USA; 6 Department of Pathology and Laboratory Medicine, Western University, London, ON, Canada; 7 Computational Biology, A.C. Camargo Cancer Center, São Paulo, SP, Brazil; 8 Rutgers University, New Brunswick, NJ, USA; 9 Universidade de São Paulo, São Paulo, SP, Brazil; 10 Institute of Applied Biosciences, Centre for Research and Technology Hellas, Thermi, 57001, Thessaloníki, Greece; 11 Department of Integrative Biology, University of California, Berkeley, CA, USA; 12 School of Mathematical Sciences, Peking University, Beijing, BJ, PR China; 13 Delft University of Technology, Delft, ZH, Netherlands; 14 Department of Biomedical Informatics, Harvard Medical School, Boston, MA, USA

**Keywords:** benchmark, environmental, SARS-CoV-2, sequencing, surveillance, wastewater

## Abstract

Wastewater-based surveillance (WBS) is an important epidemiological and public health tool for tracking pathogens across the scale of a building, neighbourhood, city, or region. WBS gained widespread adoption globally during the SARS-CoV-2 pandemic for estimating community infection levels by qPCR. Sequencing pathogen genes or genomes from wastewater adds information about pathogen genetic diversity, which can be used to identify viral lineages (including variants of concern) that are circulating in a local population. Capturing the genetic diversity by WBS sequencing is not trivial, as wastewater samples often contain a diverse mixture of viral lineages with real mutations and sequencing errors, which must be deconvoluted computationally from short sequencing reads. In this study we assess nine different computational tools that have recently been developed to address this challenge. We simulated 100 wastewater sequence samples consisting of SARS-CoV-2 BA.1, BA.2, and Delta lineages, in various mixtures, as well as a Delta–Omicron recombinant and a synthetic ‘novel’ lineage. Most tools performed well in identifying the true lineages present and estimating their relative abundances and were generally robust to variation in sequencing depth and read length. While many tools identified lineages present down to 1 % frequency, results were more reliable above a 5 % threshold. The presence of an unknown synthetic lineage, which represents an unclassified SARS-CoV-2 lineage, increases the error in relative abundance estimates of other lineages, but the magnitude of this effect was small for most tools. The tools also varied in how they labelled novel synthetic lineages and recombinants. While our simulated dataset represents just one of many possible use cases for these methods, we hope it helps users understand potential sources of error or bias in wastewater sequencing analysis and to appreciate the commonalities and differences across methods.

**Impact Statement**

Sequencing DNA from wastewater is a promising approach to identify locally circulating pathogens. Wastewater sequencing has the potential to resolve within-species genetic diversity, including viral lineages. However, deconvoluting the mixture of lineages present in wastewater based on short-read sequencing remains challenging. Numerous computational methods have been developed to do so, but it remains unclear how well they perform. Here we report a simulated dataset of SARS-CoV-2 genomes for the purposes of benchmarking deconvolution methods. We assessed nine commonly used methods, which generally performed well but varied in their handling of a novel recombinant lineage. Variation in sequencing depth and read length had little effect on the results, while the presence of a novel lineage increased the error in relative abundance predictions. While SARS-CoV-2 lineage deconvolution is generally reliable, we note potential caveats and differences among methods that should be considered by researchers and public health practitioners working with wastewater sequences.

## Data Summary

The simulated wastewater dataset is available along with the software used in the study (DOI: 10.5281/zenodo.10680922).

## Introduction

Wastewater-based surveillance (WBS) is a powerful tool to track pathogens, genes, and drugs across the scale of a building, city, or region [1]. The ongoing COVID-19 pandemic has shifted the attention to pathogen surveillance [2], and over the course of the pandemic, it has become increasingly common to track SARS-CoV-2 in wastewater [3]. SARS-CoV-2 can be tracked in wastewater due to infected individuals shedding viral particles of SARS-CoV-2 in faeces [4,7]. WBS has become a vital component of SARS-CoV-2 surveillance and is implemented across much of the world [3].

WBS of SARS-CoV-2 often relies on qPCR-based quantification of viral RNA – providing a rapid estimate of infection rates in the population [8]. A limitation of qPCR is that it provides little to no information about viral diversity – including the presence and relative abundance of genetically distinct viral lineages, including variants of concern (VOCs). Designing primers to distinguish among VOCs by qPCR [9] can be laborious and is hindered by novel viral mutations [10]. A complementary approach to qPCR is to measure viral diversity by sequencing parts, or the entirety, of the viral genomes found in wastewater [11,15].

Sequencing wastewater provides an opportunity for cost-effective epidemiological surveillance [16]. However, challenges persist both upstream and downstream of sequencing. Upstream, the quality of results may vary according to the population size contributing to the wastewater catchment area [8], variability in viral shedding [17,18] virus enrichment methods [19], and in-sewer degradation of viral-particles (e.g. pH, temperature, travel-time) [20,21]. These and other factors influence how much viral RNA is collected for sequencing [22]. Typically, viral RNA recovered from wastewater is at a lower concentration and more degraded than in clinical samples, especially outside major waves of infection [23]. Within wastewater, viral RNA makes up a relatively small fraction of nucleic acid compared to eukaryotes and bacteria [24], and PCR inhibitors in wastewater can make amplicon-based sequencing prone to uneven coverage across the genome, due to some or all amplicons failing to amplify [25]. Downstream of sequencing, mixtures of diverse viral lineages from wastewater can make it difficult to correctly assign mutations on short reads to the correct lineage [26,27]. High viral diversity in wastewater can lead to consensus genomes which are prone to chimaeras and errors, and mostly represent the dominant lineage while failing to identify rarer ones [21].

A variety of computational tools have been developed recently to address the challenges of wastewater sequencing, with the common goal of inferring the presence and (in most cases) the relative abundances of various SARS-CoV-2 lineages present in wastewater. In this benchmark study, we focus on SARS-CoV-2 whole-genome sequencing using short reads from tiled amplicons to identify specific viral lineages and their relative abundances [28]. This approach is currently more commonly used than long-read sequencing [29]. In this study, we compared the performance and output of nine commonly used computational tools, available between winter 2021 and spring 2022 in a semi-blind benchmark on simulated wastewater sequencing data. These tools differ in the precise statistical methods, criteria for inferring lineages, as well as the databases used (Table 1). The benchmark dataset consisted of simulated *in silico* mixtures of SARS-CoV-2 genomes belonging to BA.1, BA.2, Delta, Delta–Omicron recombinants, and a synthetic ‘novel’ lineage (Methods).

**Table 1.** Summary of nine approaches included in this study: input required for each approach is either FASTQ (sequencing reads), or VCF (allele frequency table relative to the Wuhan reference). The database used by each approach was divided into two categories: curated mutation profiles, where a selection of alleles present per lineage is done by a functional or phylogenetic criteria, or selected alleles from GISAID genomes, where all or some GISAID genomes are aligned to the reference genome, and alleles are selected based on allele-frequency. For VLQ, this is used in selecting a reference set for lineages when performing pseudoalignment. Database availability refers to how users can access the database, by accessing the tools’ GitHub repository or by executing a command within the tool. For VLQ, this is used in selecting a reference set for lineages when performing pseudoalignment. Database availability refers to how users can access the database, by accessing the tools’ GitHub repository or by executing a command within the tool

Tool	Package manager	Dependencies	Input	Method to estimate lineage relative abundances	Database type	Database details	Database availability	Reference
Alcov	Pip	Cutadapt, minimap2 Python Packages: Fire, numpy, pandas, scikit-learn, matplotlib, seaborn, pysam	FASTQ	Linear model: ordinary least squared	Curated mutation profiles	Cov-Spectrum	GitHub	[31]
Basic	na	blast+, PRINSEQ, fastp, bwa, picard, samtools, varscan, python3, R	FASTQ	Linear model: constrained linear model	Selected alleles from GISAID genomes	Allele-frequency>=90 %	GitHub	[30]
Freyja	Conda	iVar, samtools, UShER Python packages: cvxpy, numpy, pandas	FASTQ	Constrained (weighted) least absolute deviations	Curated mutation profiles	Marker mutations from UShER global phylogenetic tree	GitHub or; Via Freyja	[10]
Gromstole	na	cutadapt, minimap2, Python, R	FASTQ	Quasibinomial regression model	Curated mutation profiles	Cov-Spectrum	GitHub	[33]
LCS	na	Snakemake, samtools, GATK4, bwa, picard, USHER Python Packages:, biopython, pysam, pyvcf, pandas, cvxpy, ray-core	FASTQ or VCF	Log-likelihood maximization model	Selected alleles from GISAID genomes	Allele-frequency>=80 % with phylogenetic verification	GitHub or; Via LCS	[34]
Lineagespot	bioconductor	R	VCF	Average allele frequency	Curated mutation profiles	Outbreak.Info or Pangolin	na	[35]
VLQ	na	kallisto, samtools, minimap2, bwa, bbmap Python Packages: pyvcf, pysam	FASTQ	Pseudoalignment to reference genomes (Kallisto)	Selected alleles from GISAID genomes	Reference genomes selected to capture alleles with frequency>=50 %	Via VLQ	[37]
V-pipe	Conda	COJAC, ShoRAH, LolliPop Python Packages: pysam, pandas, numpy, pyyaml, strictyaml, requests, click, poetry-core	FASTQ	Linear model: constrained linear model (Lollipop) and Dirichlet process mixture model (ShoRAH)	Curated mutation profiles	Cov-Spectrum and UKHSA Genomics Public Health analysis variant-definitions	Via COJAC	39–42
Pipes	na	bowtie2, R (ape)	FASTQ	Expectation-maximization algorithm	Selected alleles from GISAID genomes	Calculated phylogenetic internal nodes for each lineage	Via Pipes	[36]

Our goal was to assess how each tool performs lineage identification and quantification of mixtures of SARS-CoV-2 lineages, as would be found in wastewater, in comparison to a ‘Basic’ approach. The Basic approach was a preliminary attempt that showed promise in the deconvolution of lineages from wastewater samples before the development of more sophisticated tools [30], highlighting the progress that has been made since. We used simulated wastewater samples, and while true wastewater samples likely contain more diverse mixtures than simulated here, we believe our study provides a logical foundation for more complex benchmarking. To generate sequencing data resembling typical wastewater sequences, we simulated random sequence errors, variable depth of coverage and read length, amplicon drop-outs, and the presence of an unknown lineage. These conditions are meant to reflect real-world financial and operational constraints on sequencing methods and effort. While the dataset is meant to reflect these considerations, it remains a model use case that does not necessarily reflect the complexity of real-world data. We also acknowledge that this is not a comprehensive comparison of all available approaches, nor can this be considered a fully quantitative or unbiased comparison, not least because the developers of each tool were given the benchmark dataset and ran their tool as they saw fit, according to their own best practices. We found that in general all tools performed their function very well and that their results were largely robust to such challenges as low sequencing depth or shorter read lengths, thus raising confidence in VOC calling, no matter the specific sequencing conditions and tool used. The tools included in the benchmark test are described in detail in their respective publications. A brief description of each tool follows.

### Alcov

A python package designed to leverage both unique, lineage-specific mutations, and shared, non-lineage specific mutations [31]. ‘Double-counting’ of shared-mutations is avoided by performing a lineage prediction *a priori* to estimate expected mutation frequencies in each sample. With an expected, and observed mutation frequency, a most likely frequency is determined by minimizing the error between predicted and expected mutation frequencies. For lineages in which there are at least 100 sequences, Alcov uses lineage-defining mutations selected from cov-spectrum [32] that are present in >90 % of lineages.

### Basic

A preliminary pipeline devised to analyse wastewater sequences from variant lineages during WBS in Québec early during the pandemic (between March 2020 and July 2021 [30]). The pipeline is composed of two components: variant calling of single-nucleotide variants that pass coverage and quality thresholds, followed by post-variant-calling analysis. Post-variant-calling calculates relative abundances of lineages with a constrained linear model to fit the lineages’ signature mutations (at least three per lineage) at allele-frequencies over 90 %. This pipeline was designed and tested before the first Omicron wave. It also only considers minor alleles in wastewater samples at a frequency of 25 % or higher, making it less sensitive to rare variants and more attuned to confidently identifying common lineages.

### Freyja

A suite of analysis tools for real-time wastewater genomic surveillance. Freyja includes a ‘demix’ function that enables users to estimate the relative abundance of virus lineages in a mixed sample, such as wastewater [10]. This method encodes genomic sequencing of virus mixtures using single-nucleotide variant (SNV) frequencies and leverages unique mutational barcodes for each lineage (assembled using the most recent UShER global phylogenetic tree). To recover relative lineage abundances from a mixture, the barcodes and SNV encodings are integrated in a mixture model formulation that is solved using a constrained, depth-weighted least absolute deviations approach. The use of depth weighting confers robustness to limited sequencing coverage, a common feature of wastewater samples. Freyja is written in Python, has a command line interface, and is available via conda.

### Gromstole

A lightweight pipeline [33] that consists of Python and R scripts to pre-process samples and extract the read coverage and mutation frequencies per nucleotide site for each sample. It uses a curated list of lineage-specific mutations. The frequency of a lineage in the sample is estimated by assuming the frequency of each associated mutation is an independent outcome. A quasibinomial regression model is used to compute 95 % confidence intervals for each frequency estimate. The pipeline is designed to be lightweight and minimize the number of dependencies required. It was not designed to call novel variants or recombinant forms of SARS-CoV-2. To mitigate the problem of numerous shared mutations among lineages, the tool uses a curated list of lineage-specific mutations to exclude mutations that occur at substantial frequencies in other (‘off-target’) lineages, with the concession of substantially reducing the number of mutations that can be used to estimate lineage frequencies.

### LCS

A viral genome deconvolution approach based on relative frequencies of polymorphisms found in known SARS-CoV-2 variants [34]. The reference database is built, first, with pre-defined SARS-CoV-2 lineages assigned to variant groups (VGs) according to the currently tracked variants defined by the World Health Organization. Next, manually curated genome designations from the Pango Lineage Designation Committee are assigned to the corresponding VGs. These genomes, whose sequences are available in the GISAID database, are mapped to the SARS-CoV-2 reference genome and polymorphic sites with allele frequency greater than 80 % in at least one VG are selected. Based on these markers, the method fits a mixture model to pools of SARS-CoV-2 samples, considering the relative frequencies of polymorphisms found in each pool to obtain a maximum *a posteriori* estimate of the relative contributions of SARS-CoV-2 variants to the pool.

### Lineagespot

A bioconductor R-package [35] that uses VCF files, which contain all the nucleotide (and corresponding amino acid) changes identified in a sample, along with a file containing all lineage-assignment mutations. Lineagespot uses identified mutations grouped and assigned to the respective SARS-CoV-2 lineages and computes a number of metrics (such as the average allele frequency, and the minimum non-zero allele frequency). It attempts to determine the presence of a specific lineage (specified as input to the tool) by calculating the likelihood of its existence in the sample, rather than its relative abundance. This benchmark exercise, which assesses the ability of each tool to infer lineage relative abundances, therefore extends beyond the original scope of Lineagespot.

### Pipes *et al.* 2022 (‘Pipes’)

A method [36] that builds a reference database, constructs a read ×haplotype mismatch matrix, and then uses an expectation-maximization (EM) algorithm to obtain the maximum-likelihood estimates of the proportions of different haplotypes in a sample. The reference database is constructed using a phylogenetic imputation approach.

### Viral lineage quantification (VLQ)

A pipeline [37] that differentiates between lineages and sub-lineages based on a reference set that can be tailored to the location and time of sampling. VLQ consists of three steps: first, it constructs a reference set consisting of one or more full genome sequences per SARS-CoV-2 lineage of interest, selected to represent local variation. Then, VLQ uses kallisto [38], an algorithm for RNA transcript abundance quantification from RNA-Seq data, to predict the relative abundance of each reference sequence. Finally, VLQ performs basic post-processing: background noise is removed by filtering out predictions below a user-specified minimal abundance level and subsequently the predicted abundance for sequences from the same lineage (or VOC) are summed to obtain the total abundance per lineage (or VOC).

### V-pipe

In the context of this benchmark, V-pipe refers to a workflow developed in the Beerenwinkel Lab at ETH Zurich [39], which includes components developed for wastewater sequencing data analysis: COJAC [40], LolliPop [41] and ShoRAH [42]. V-pipe pre-processes samples, and COJAC searches for co-occurrences of variant signature mutations on individual PCR amplicons. For the detected variants, relative abundances are quantified using LolliPop by simultaneously smoothing and deconvolving the observed mutation frequencies in sequencing reads into relative abundances of the variants and providing confidence bands. ShoRAH identifies unknown lineages by a Bayesian nonparametric read clustering method, which denoises the sequencing data and reconstructs local haplotype sequences. Inferred local haplotypes with mutations different from any known variants are considered signatures of new variants.

## Results

The benchmark test dataset consisted of 100 simulated wastewater samples containing either single lineages or mixtures, at high or low coverage, and with long- or short-read lengths (Methods, Table S1, available in the online version of this article). Samples were provided to the developers of each tool and run according to their own best practices on their own systems. Each team was aware that samples would contain BA.1, BA.2 and Delta sequences, but were not told the composition of each sample. We evaluated how each tool identified lineages and how well they estimated relative abundance.

**Fig. 1.**
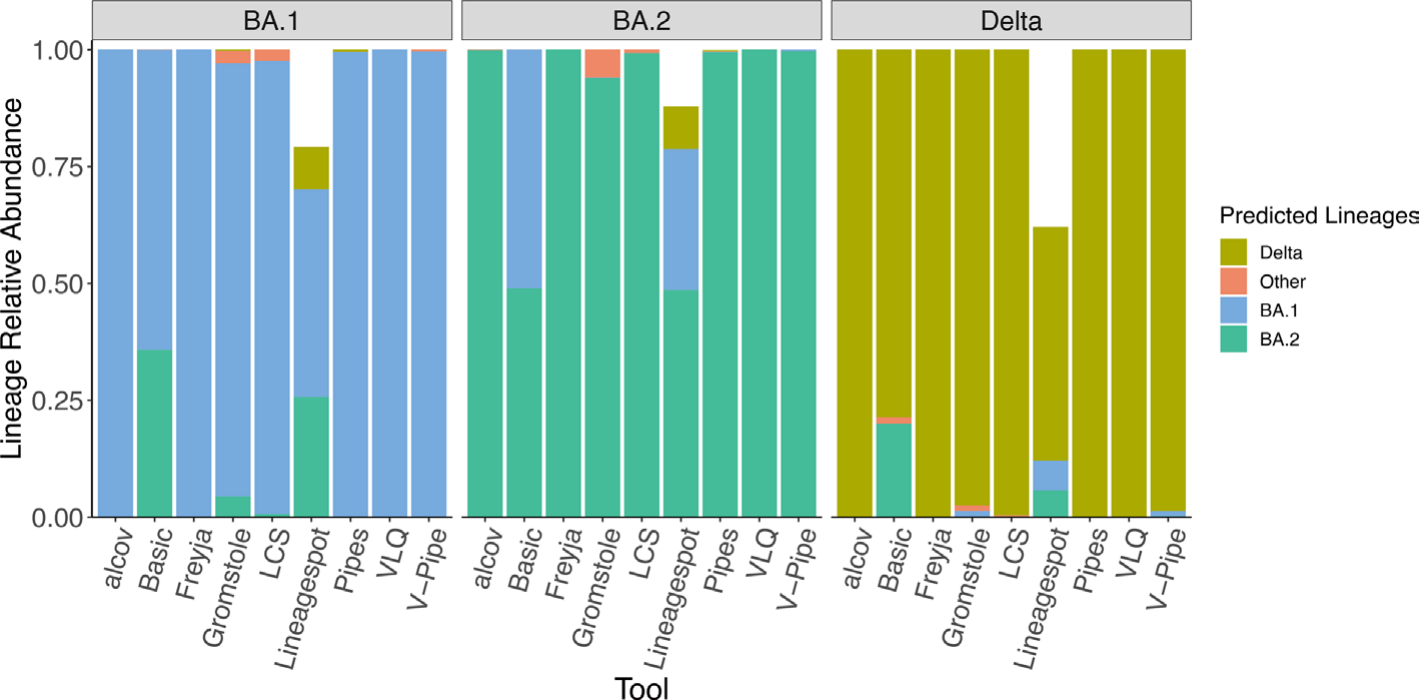
Inference of BA.1, BA.2 or Delta in single-lineage samples. Relative abundance of single-lineage predictions for samples of BA.1, BA.2, Delta with simulated 150 bp reads and low coverage (37.5xmean depth). ‘Other’ is defined as lineage calls that are not either BA.1, BA.2 or Delta.

We first considered samples that contained only a single lineage of either BA.1, BA.2, or Delta, at low coverage (37.5×mean depth) and short 150 bp reads (Fig. 1). Most of the tools correctly identified the true lineage, at close to the expected 100 % frequency (Fig. 1), with the exception of Lineagespot, which is designed to infer the presence/absence of a lineage rather than its abundance (that is, it provides only the absolute minimum guaranteed level of presence). ‘False positive’ lineages were sometimes inferred, typically at low frequency. The exception was the Basic approach which could not distinguish BA.1 and BA.2 (Fig. 1).

**Fig. 2.**
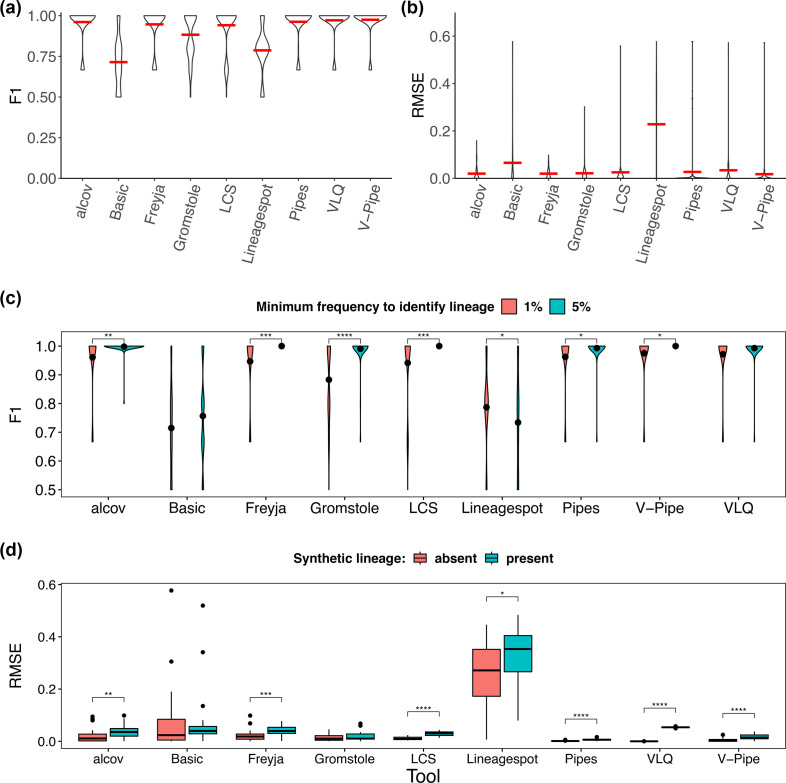
Lineage identification and quantification in single- and mixed-lineage samples. Based on all 100 simulated samples, we calculated (**a**) F1-scores and (**b**) root-mean-square error (RMSE) for each tool. Horizontal red lines show the mean. Lineage presence is defined by detection at ≥1 % abundance in a sample. (**c**) Comparison of F1-scores with lineage presence defined by≥1 % or ≥ 5 % abundance (black point shows the mean F1). (**d**) Comparison of relative abundance estimates, RMSE, between mixed-lineage samples with a synthetic lineage present (*n*=22) or absent (*n*=36). * Wilcox *p*-adjusted (BH)<0.05.

Next, we considered mixed-lineage samples and compared the ability of each tool to identify the correct lineages (F1-score) and estimate their relative abundances (root-mean-square error; RMSE). With some variation, tools were generally able to identify the correct lineages present (Fig. 2a) and estimate their relative abundances (Fig. 2b), i.e. most tools had consistently high F1-scores and low RMSE-scores. Exceptions were Lineagespot and Basic, which had relatively low F1-scores and higher mean RMSE across simulation parameters. Lineagespot was designed to determine the probability of a lineage being found in a wastewater sample and is being extended beyond its original scope in this study to include relative abundance predictions. We see this reflected in its high RMSE (Fig. 2b). This also led to a low F1-score (Fig. 2a), likely because relative abundance cut-offs were used to define a lineage’s presence, leading to high false-positivity rate (low-specificity, Fig. S1) but low false negativity (high-recall, Fig. S1). Basic’s relatively low F1 and higher RMSE score are unsurprising, given its preliminary, and relatively naive design (developed and tested on lineages pre-Omicron, and for detecting lineages well above 1 % relative abundance).

Lineage detection in a sample is intuitively more likely if the lineage is present at a higher frequency. In our study, a lineage was considered present in a sample if it was found at 1 % or more. However, a previous study set the limit of detection at 5 % [10]. We compared F1-scores at 1 % vs. 5 % cut-offs for detection and found that most tools had higher F1-scores when the 5 % cut-off was used (Fig. 2c). Despite fewer possible ‘true-positives’, the higher F1-score is driven by a decrease in both ‘false-positives’ and ‘false-negatives’, or increased precision and/or recall respectively (Fig. S2). We also compared lineage identification and relative abundance quantification between samples with different parameters: high or low coverage, 150 or 250 bp reads, with or without amplicon drop-outs, and with or without the presence of a synthetic lineage. Sequencing always comes with a background error rate, and amplicon-based sequencing can be sensitive to amplicon dropouts when mutations interfere with primer binding. We found no significant differences between samples with and without amplicon dropout, low-coverage, or short-reads in lineage identification or estimates of relative abundances (Fig. S3). However, the presence of an unknown lineage had a significant effect on lineage quantification (Fig. 2d). Adding an unknown lineage increased RMSE consistently across tools. The magnitude of this effect was relatively small but could impact lineage deconvolution in more complex background conditions. Together, these findings highlight that lineage calling can be confidently performed even with relatively low sequencing depth and short-read lengths, but that lineages predicted to be present below 5 % relative abundance should be interpreted with caution.

**Fig. 3.**
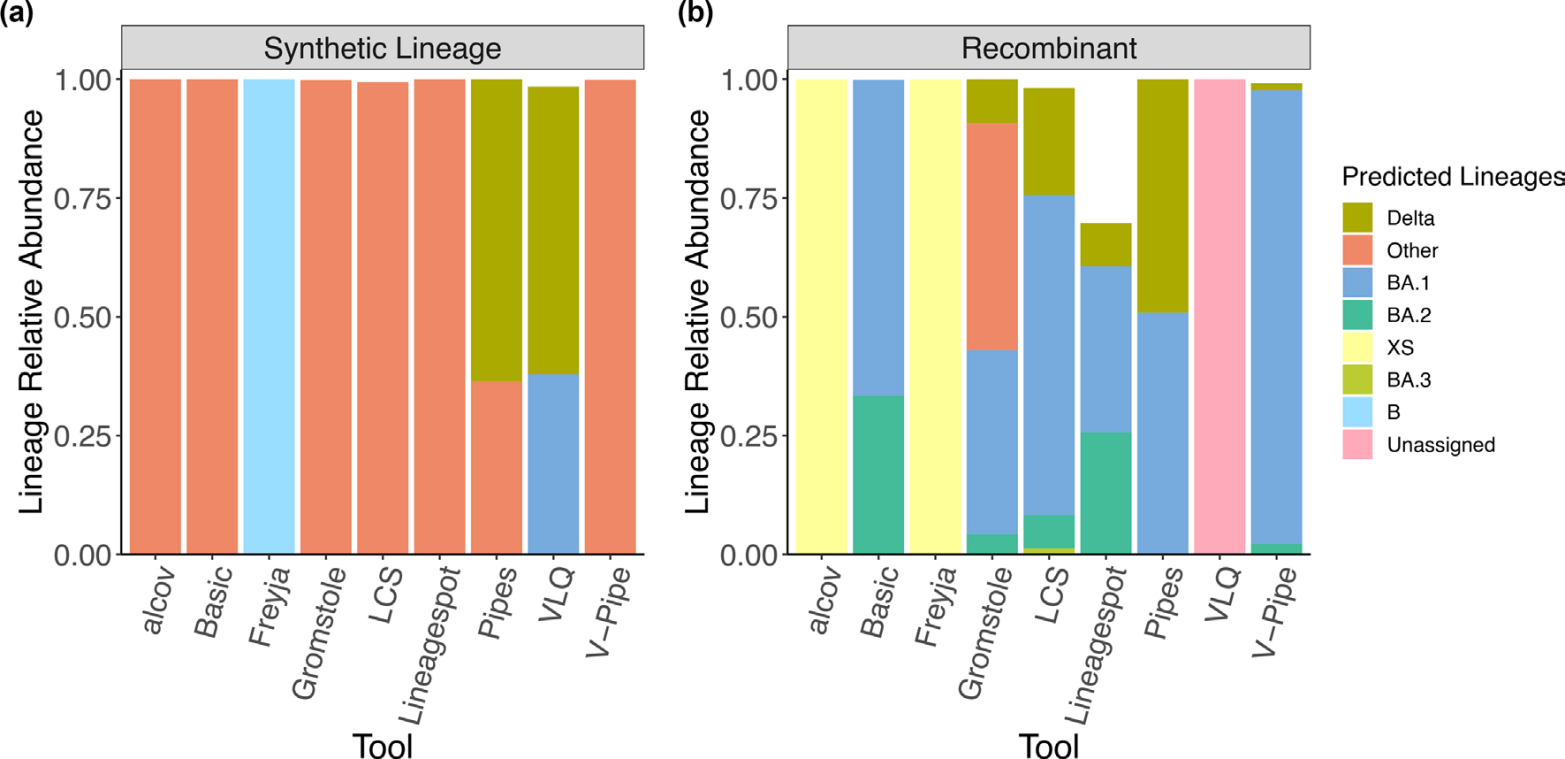
Identification of unknown lineages in simulated wastewater sequences. (**a**) Each tool was applied to a sample containing a simulated synthetic lineage sequenced with 150 bp reads and low coverage. The synthetic lineage is the Wuhan reference genome with the addition of 50 non-lineage defining mutations. (**b**) Each tool was applied to a sample containing a simulated recombinant of Delta:BA.1.1 from low-coverage 250 bp reads. Only predictions of lineages>1 % are represented in the barplot.

Having explored the performance of tools within their intended use of detecting known lineages, we considered more challenging tasks of identifying unknown or recombinant lineages. First, we asked how the tools interpret a synthetic lineage which is not present in any of the databases. We simulated this synthetic lineage based on the Wuhan reference genome background, adding 50 mutations previously observed in real genomes, but never rising to high frequency nor appearing together in the synthetic combination (Methods). This lineage could represent a newly evolved or introduced lineage. Most tools (Alcov, Basic, Gromstole, LCS, Lineagespot, V-pipe) identified the reads as belonging to ‘Other’ lineages rather than attempting a designation such as BA.1, BA.2, or Delta (Fig. 3a). Freyja, which employs the UShER phylogenetic tree for lineage-identification, placed the synthetic lineage near the root as a deep-branching ‘B’ lineage (Fig. 3a). There were also some instances of ‘false-positive’ identification of Delta or BA.1 (Pipes and VLQ) (Fig. 3a). Together with the observation that the synthetic lineage can weakly but consistently add error to relative abundance estimates of known lineages (Fig. 2d), these results highlight the challenges associated with handling novel lineages in mixed wastewater samples.

Second, we explored how each tool dealt with a recombinant of Delta and BA.1.1. Several tools (Pipes, Gromstole, LCS, Lineagespot, Basic, and to a lesser extent V-pipe) split their lineage calls between the parent lineages, Delta and Omicron (Fig. 3b). Freyja and Alcov called it ‘XS’, which is a Delta:BA.1 recombinant, while VLQ left it unassigned.

## Discussion

Prior to the SARS-CoV-2 pandemic, wastewater-based surveillance had been an important part of the epidemiological polio surveillance and eradication effort [43]. The discovery that one could differentiate between vaccine and non-vaccine-derived polio virus without culturing the virus from environmental samples [44] showed that it should also, in principle, be possible to detect and estimate the relative abundances of different lineages of SARS-CoV-2 from wastewater [45]. Detecting and quantifying viral lineage relative abundance from a mixed wastewater sample is greatly facilitated by clinical genomic protocols [46,47]; however the processing of this sequence data is not trivial, providing the motivation for this study.

Nine different teams, across six different countries, developed distinct approaches to distinguish SARS-CoV-2 lineages from wastewater. We acknowledge that this was not an exhaustive set of methods, and new methods have been developed since our project was initiated. The common goal of all approaches is to identify SARS-CoV-2 lineages and estimate their relative abundance. Lineagespot, the exception, was not designed to infer the relative abundances directly. Each team made assumptions during the development of their approach as to how, and when, their tool would be used, and this benchmark did not aim to reflect all possible scenarios. For example, should all SARS-CoV-2 genomes circulating globally be used in the set of lineage-defining alleles, or only a locally/temporally matched subset? Are lineage-defining alleles curated based on functional characteristics, phylogenetics, or frequency within the population? What type of sequence pre-processing is required, and/or optional? Should all alleles observed in a lineage be included? If not, what criteria should be used in selecting lineage-defining alleles? What priorities should be favoured in selecting a model of lineage deconvolution for mixed-viral samples? Our simulated benchmark dataset represents one common public health use case [48], and addresses technical considerations upstream of bioinformatic analysis (i.e. read length, and minimum genome coverage), but falls far short of evaluating all use cases for the individual tools.

This study was initiated when most of the approaches were still under active development, which made running each tool locally by one user impractical. Instead, the benchmark was implemented as a semi-blind trial: each team knew to expect a mixture of Delta and Omicron lineages, but not a synthetic ‘novel’ sequence or a simulated recombinant genome. Each team ran their own method on the simulated data and was thus able to make some ‘expert’ decisions that might not be apparent to a naive user. A more stringent comparison would have one group run all the methods independently, thus more closely simulating the experience of a new user running a tool ‘out of the box.’ Such an approach would allow a more direct measure of user-friendliness, reproducibility, and compute time.

All approaches require the identification of lineage-defining alleles for lineage detection, and the steps involved vary among approaches, making side-by-side comparisons challenging. We classified tools into two categories of reference databases: mutation lists curated by the team or based on the allele frequencies within a lineage in the GISAID database (Table 1). Genomes in the benchmark came from multiple different countries, which means that approaches that build reference sets based on a subset of genomes from GISAID (e.g. VLQ) could not leverage the metadata (i.e. time or geographic location) for selecting suitable reference sequences per lineage but instead included the reference genomes used in building the benchmark dataset and others present in GISAID. While lineage-agnostic analyses of wastewater were outside the scope of this benchmark, these approaches have shown promise in identifying cryptic lineages of SARS-CoV-2 in wastewater [49].

We found that most approaches identify lineages and estimate their relative abundances as intended. A surprising finding was that tools could detect lineages at abundances down to 1 %, below the level of 5 % used previously [10]. Despite the ability of tools to correctly classify mixtures with 1 % lineages, increasing the cut-off for detection to 5 % reduced ‘false-positives’, and significantly improved F1-scores for most tools. Lineages detected below 5 % should therefore be interpreted with caution. We also explored several factors that could impact the utility of WBS: genome coverage, read-length, and amplicon drop-out. None of these factors significantly affected lineage calling or relative abundance estimates, with the caveats that amplicon drop-out was only simulated for three of 100 amplicons. In addition, amplicon drop-out and read length were tested with smaller subsets of samples, 26, and 48 respectively. Together, our results suggest that reducing coverage and read length (to a point) could help cut costs while preserving most of the desired lineage information. These considerations could help make WBS epidemiologically informative and cost-effective, particularly in resource-poor settings [50].

With the goal of testing how tools dealt with a novel lineage, we simulated a synthetic lineage with non-lineage defining mutations. As none of these mutations are lineage-defining, this could also be viewed as simulating recent *de novo* mutations in the population, or common sequencing errors. Most tools (Alcov, Basic, Freyja, Gromstole, LCS, Lineagespot, Pipes, V-pipe) could correctly identify the presence of a non-Omicron, non-Delta lineage in the sample. The presence of a synthetic lineage in mixed samples weakly but significantly impacts the relative abundance estimation for a number of tools (Alcov, Freyja, LCS, Lineagespot, Pipes, VLQ, and V-pipe). We also investigated how tools handled an unclassified recombinant of Delta–Omicron. Faced with an unclassified recombinant genome, tools either identified the two parental lineages, acknowledged it was an unknown lineage, or found the closest known lineage. While recombinant and synthetic lineage identifications fall beyond the intended scope of most tools, they give us some idea how tools might behave when faced with the diversity of lineages present in a real wastewater sample.

The simulated dataset described here was developed in 2021 and the nine teams applied their methods to the data in 2022. This study therefore reflects the questions we had at the time. Since then, the recombinant XBB (Nextstrain clade 22F) has risen to global abundance, and convergent mutations have made it increasingly challenging to disentangle lineages. As we write, SARS-CoV-2 genome sequencing has been deployed in wastewater from across the globe (e.g. USA, Canada, Uruguay, Japan, Italy, India, Spain) to augment surveillance efforts during Gamma, Delta, and Omicron (BA.1 to XBB/BQ) waves [12,13, 27, 28, 51]. The uptake of wastewater sequencing data to bring lineage-level information to WBS highlights the importance of benchmarking and developing standards. Such standards have emerged for clinical sequencing of SARS-CoV-2 [52] and we hope that our work will inspire further efforts towards standards for wastewater sequence data. Just as efforts to quantify and sequence SARS-CoV-2 from wastewater built on the knowledge gained through polio surveillance efforts, WBS is now being applied to other pathogens such as influenza, metapneumovirus, parainfluenza, respiratory syncytial virus (RSV), and rhinovirus [53,54]. Wastewater has mostly been sampled and analysed in cities, but there are efforts to expand surveillance to airplanes [55] and more rural communities [56]. As new sampling schemes [57,58], sequencing technologies [59,60], and computational methods [61] are developed, it is essential to understand their behaviour using simulated data.

## Methods

### Benchmark dataset

We randomly selected 10 high-quality genomes, from which we simulated short sequence reads including random sequencing errors, as described below. Each genome belonged to Delta, BA.1, or BA.2 lineages chosen from among the cov-lineages pango-designation lineages (github.com/cov-lineages/pango-designation/blob/master/lineages.csv?; accessed March 2022) and were downloaded from GISAID [62] (Table S2). Genomes were from human-derived samples, as environmental samples uploaded to GISAID still undergo a consensus assembly, which could introduce chimaeras. The genomes were selected randomly, with no bias toward genomes that were ‘representative’ of their lineage. All genomes were verified not to contain long stretches of Ns.

Recombinant genomes, common in betacoronaviruses, arise from distinct viral lineages that recombine during viral replication, resulting in a new viral lineage that shares genome segments from both parental genomes [63]. Not all recombinants are identified [64] or establish onward transmission [65] and therefore receive a lineage-distinction. For the benchmark dataset we selected four genomes of closely related recombinants, that do not have a lineage distinction, and appear to have arisen from the recombination between AY.119.2 (Delta) and BA.1.1 (Omicron) [66].

To create a synthetic genome, we started from the reference genome hCoV19/Wuhan/WIV04/2019, to which we added 50 mutations. These mutations were randomly selected from those present at minor frequencies (10 % or less) in Alpha lineage genomes in the GISAID multiple sequence alignment published on 27 March 2022. To be chosen, mutations needed to be observed in at least 1000 genomes and were double-checked to ensure that they were not defining for any known PANGO constellation (https://cov-lineages.org/constellations.html). Mutations were excluded if they caused a stop codon or were located in the intergenic area of the genome.

Amplicons were created by cutting out the ARTIC v4.1-based fragments from the genome using seqkit [67]. To simulate uneven coverage across genomes we also created a BA.1 fragment set in which three PCR amplicons (6, 55, 74) were dropped from all BA.1 genomes. Artificial reads were created using insilicoseq [68]. We created custom error models based on wastewater samples sequenced with a read length of either 150 or 250 bp using bwa mem (v. 0.7.17) and samtools (v.1.12). The *in silico* reads were subsequently randomly subsetted and combined to create 100 different samples representing single strains, as well as mixtures of two or three strains with differing coverage and read lengths. Low coverage samples with 250 bp reads included 4000 reads; 150 bp read samples included 7500 reads. High coverage samples with 250 bp reads included 80 700 reads; 150 bp read samples included 150 000 reads. A total of 100 samples were generated, varying by the lineages present, their relative abundances, read length, and coverage (Table S1). Samples varied in mixtures of lineages as follows; one lineage (*n*=22), two lineages (*n*=62), and three lineages (*n*=16); read lengths; 150 bp (*n*=24), 250 bp (*n*=76); coverage; high-coverage (*n*=50), low-coverage (*n*=50); number of reads; 4000 (*n*=38), 7500 (*n*=12), 80 700 (*n*=38), or 150 000 (*n*=12). Lineages were found in relative abundances ranging from 1–100 %. Of these different ratios, with 16 samples containing a lineage at 1 % frequency (Table S1).

### Tools

#### Alcov

Preprocessing for Alcov was done with the alcov-prep.py script which uses cutadapt (4.1), minimap2 (2.24 .r1122), and samtools (1.7) to trim adapter sequences and map reads to the reference genome. Alcov was then run on each sample to generate lineage predictions using the command ‘alcov find_lineages --min_depth=10 --unique=False --csv=True samples.txt’ with constellation files updated on 20 June 2023.

#### Basic

The variant calling step was run with default parameters: minimum coverage of 50× and a conservative minimum allele frequency of 25 % based on findings previously reported [30]. Post-variant-calling was modified for this benchmark to include BA.1, BA.2, and BA.3, not included in the original pipeline, and updated the mutation prevalence file built using multiple-sequence-alignment on all genomes. Mutations with prevalence of >=90 % for each VOC investigated here (Alpha, B.1.351, Gamma, Delta, Lambda, Mu, BA.1, BA.2, BA.3) based on the GISAID msa from 27-03-2022. This included 300 mutations associated with lineages including Alpha (28), B.1.351 (19), Gamma (32), Delta (25), Lambda (25), Mu (26), BA.1 (42), BA.2 (62), and BA.3 (41). Of these, 253 were unique mutations with 40,56,18 associated with BA.1, BA.2 and Delta, respectively.

#### V-pipe

Reads were processed using V-pipe using the SARS-CoV-2 virus base config, which sets the aligner to bwa and the reference genome to the NCBI reference sequence NC_045512.2. V-pipe performed raw read quality filtering and alignment, and computed per-sample statistics, such as the per-position base counts. Detection of genomic variants in the mixed samples was performed using COJAC. For generating variant definitions, two different sources were used. A set of exhaustive lists of mutations by querying Cov-Spectrum for the Omicron variants BA.1 and BA.2, and Delta (B.1.617.2). For the Delta variant, mutations were imported from the mutation signature reported by Public Health England Genomics under the name ‘empathy-serve’. For COJAC and LolliPop, this ‘empathy-serve’-derived definition of Delta was used in addition to the exhaustive Omicron definitions. For COJAC, selected relevant amplicons were used covering combinations of mutations exclusive to B.1.617.2*, BA.1*, and BA.2*, by manual inspection, and then confirmed by querying Cov-Spectrum for the frequency at which these mutation combinations are found in the background. COJAC was run on the alignments produced by V-pipe on amplicons carrying cooccurrences of two or more mutations from the lists. For the detected variants, we quantified their relative abundances using LolliPop. LolliPop was run using the soft l1 loss, with default l1/l2 loss breakpoint at 0.1. LolliPop is tailored to analysing time series wastewater sequencing data, and thus incorporates joint temporal kernel smoothing and deconvolution. However, in this benchmarking study the data was not a time series and hence smoothing was avoided by using a Gaussian kernel with bandwidth 10^−17^. ShoRAH was used to identify unknown lineages. Reads were merged paired-end using flash (version 1.2.11), before realigning to the reference using bwa-mem. The alignment was then tiled into the 98 ARTIC amplicon regions. For each amplicon region, local haplotypes were reconstructed by sampling from a Dirichlet Process Mixture Model. A Gibbs sampler was used to derive estimates of haplotypes and their frequencies in each amplicon region. The sampler was then run for a minimum of 300 000 steps or 15 times the read coverage. Only the last 100 sampling iterations for the final estimates were kept. To present the results, SNV calls were searched for each sample for mutations not found in any of the definitions (using the exhaustive mutation lists). The samples were sorted by descending number of such new mutations.

#### Freyja

Freyja preprocessing was performed by aligning paired-end FASTQ data to the reference genome with minimap2, followed by sorting and indexing of the resulting bam file with samtools. Bam files were then processed with the standard freyja variants and freyja demix commands (Freyja v1.3.7, doi:10.5281/zenodo.6585068), using the lineage barcodes from 2 June 2022.

#### Gromstole

FASTQ data were trimmed using cutadapt discarding trimmed reads below a minimum length of 10 (default) nucleotides. Then Minimap2 maps reads to the SARS-CoV-2 reference genome (GenBank accession NC_045512) using Gromstole with default settings. Constellations were v.0.1.10 with modification, which is forked cov-lineage constellations (5 May 2022) with shared mutations between BA.1, BA.2.75, B.1.617.2, BA.4 and BA.5 removed, and BE.1 and BQ.1.1 added.

#### LCS

FASTQ files were loaded into LCS and processed with default settings and pre-generated markers (v1.2.124).

#### Lineagespot

Cleaned reads were mapped to the SARS-CoV-2 reference genome (Wuhan variant, NC_045512), using the Minimap2 tool. From this process, only the paired-end sequences were retained, while any other (unmatched, multiple mappings, etc.) were removed. In the next step, two different computational workflows were employed corresponding to the two different sequence lengths created by the NGS platforms. The first approach required primer removal, in which any primer sequences are excluded using the iVar tool. The final sequences are then remapped to the same reference genome. For the second approach, primer trimming and remapping to the reference genome is not applied. Finally, in order to be able to detect low-frequency mutations, the freebayes variant caller was used with a low mutation frequency parameter of 0.01. Ultimately, all identified mutations were annotated using the SnpEf tool and the NC_045512.2 (version 5.0) database. The proposed variant calling pipeline (with all set parameters) is provided through the GitHub repository (https://github.com/BiodataAnalysisGroup/lineagespot/blob/master/inst/scripts/raw-data-analysis.md).

The annotated VCF files, corresponding to each sample from preprocessing, were given as input to lineagespot, together with the definition files of the target SARS-CoV-2 lineages’ profiles of lineages included in study (i.e. BA1, BA.2, Delta), as these were retrieved from outbreak.info. lineagespot ran for all samples altogether and the resulting tables were merged into a single file. Finally, the assignment of each sample to one of the provided options was performed by a semi-supervised assessment of each entry, in particular across samples that exhibited similar profiles (and metrics) across multiple definitions.

#### VLQ

Reference set construction was performed without temporal or geographical parameters due to the nature of the benchmark dataset, instead the reference set was created to be representative of all SARS-CoV-2 genomes in GISAID since the beginning of the pandemic until the time of download (12 June 2022). In total 2527 sequences were selected using VLQ preprocessing (step 1), and the 34 reference genomes of the benchmark. Following reference set construction, the VLQ pipeline (steps 2 and 3) was run using default parameter settings.

#### Pipes *et al*

The reference database was built using the SARS-CoV-2 imputation command using 11 238 internal nodes from the GISAID SARS-CoV-2 global phylogeny of 7 603 548 genomes posted on 1 May 2022. Sequences from the internal nodes were estimated based on the maximum of the posterior probability of each nucleotide. The mismatch matrix was constructed using the eliminate strains programme followed by EM_C_LLR.R (R-script) to run the EM-algorithm on the mismatch matrix to obtain the maximum likelihood estimates of the proportions of different haplotypes in a sample.

Overall performance was tested based on the ability to identify lineages and estimate their relative abundance. Lineage identification did not involve distinguishing finer-grained PANGO-lineages (e.g. BA.2.1.7 vs BA.2.23.1), a feature not supported by all approaches. For the sake of comparison, we focused on broader-scale distinctions among WHO-designated VOCs (e.g. Delta vs. Omicron) or Nextstrain Clades (e.g. BA.1/21K vs BA.2/21L). To allow for comparisons between approaches, we collapsed finer-scale lineage predictions, if present, into broad categories of Delta, BA.1, and BA.2.

For tools/approaches that output relative abundances that do not sum to one, we classified the remaining relative abundance as ‘Other’ as they represent unclassified reads (Basic, Gromstole, alcov, LCS). For tools that predicted sublineages (alcov, Basic, freyja, LCS and VLQ) of Delta, BA.1, or BA.2 we merged lineage abundances into their parent lineage, with remaining lineages classified as ‘Other’. Pipes performed the merging of haplotypes and submitted the main-lineage distinction. The exception was for predictions used in evaluating the single lineage assignment for the synthetic and recombinant genomes (Fig. 3) where the submitted lineages were kept for the tools: alcov, Basic, freyja, LCS, and VLQ. In this instance we merged lineages only that belonged to one of the lineages in the Outbreak.info curated lineage list (5 May 2023).

A lineage was considered present when identified above a relative abundance cut-off of either 1 % or 5 %. We calculated the following metrics based on their standard definitions:

$$Accuracy:\frac{TP+TN}{TP+TN+FP+FN}\;Precision:\frac{TP}{TP+FP}\;Recall:\frac{TP}{TP+FN}$$

$$Specificity:\frac{TN}{TN+FP}\;F1-score:\frac{2(Recall*precision)}{Recall+precision}$$

Where *TP = True Positive, TN = True Negative, FP = False Positive, FN = False Negative*

## supplementary material

10.1099/mgen.0.001249 Uncited Supplementary Material 1.

## Abbreviations

BH: Benjamini-Hochberg procedure
qPCR: quantitative polymerase chain reaction
RMSE: Root-mean-square-error
SARS-CoV-2: Severe Acute Respiratory Syndrome Coronavirus 2
SNV: Single-nucleotide variant
VG: Variant Group
VOC: Variant of Concern
WBS: wastewater-based surveillance
