# Supplementary material for: Tracking SARS-CoV-2 variants of concern in wastewater: an assessment of nine computational tools using simulated genomic data
Source: Microb Genom. 2024 May 24;10(5):001249. doi: 10.1099/mgen.0.001249 (PMC11165662; doi:10.1099/mgen.0.001249)
Supplement: Uncited Supplementary Material 1. [file mgen-10-01249-s001.pdf]

Supplementary Materials

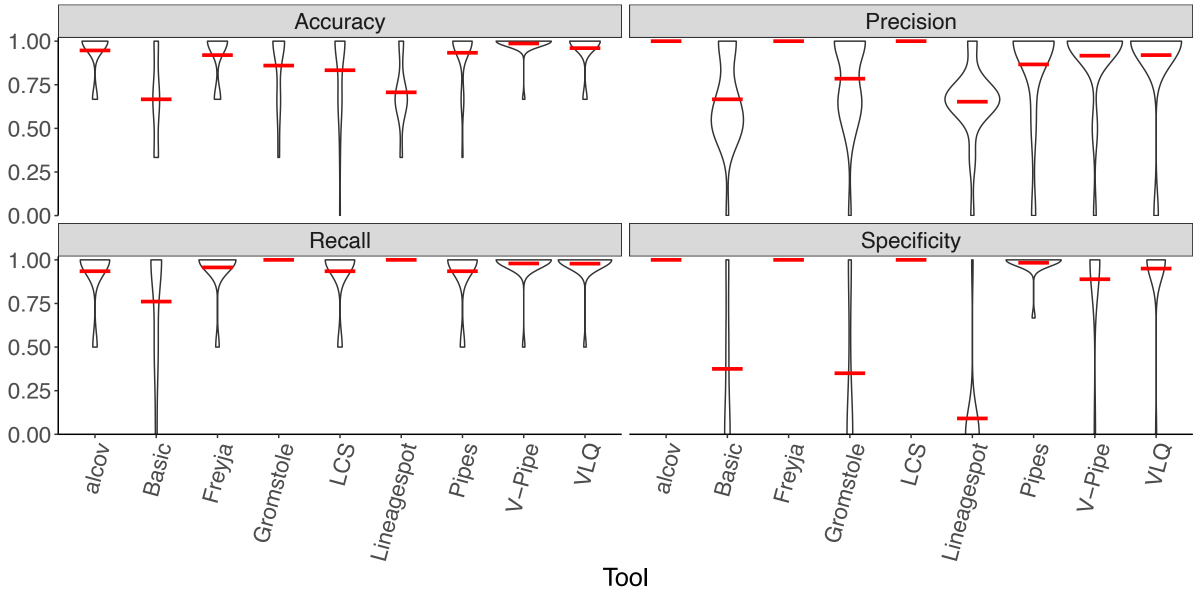

Figure S1. Lineage identification in single- and mixed-lineage samples. Based on all 100 simulated samples, we calculated accuracy, precision, recall, and specificity (Methods). Horizontal red lines show the mean. Lineage presence is defined by detection at ≥ 1% abundance in a sample.

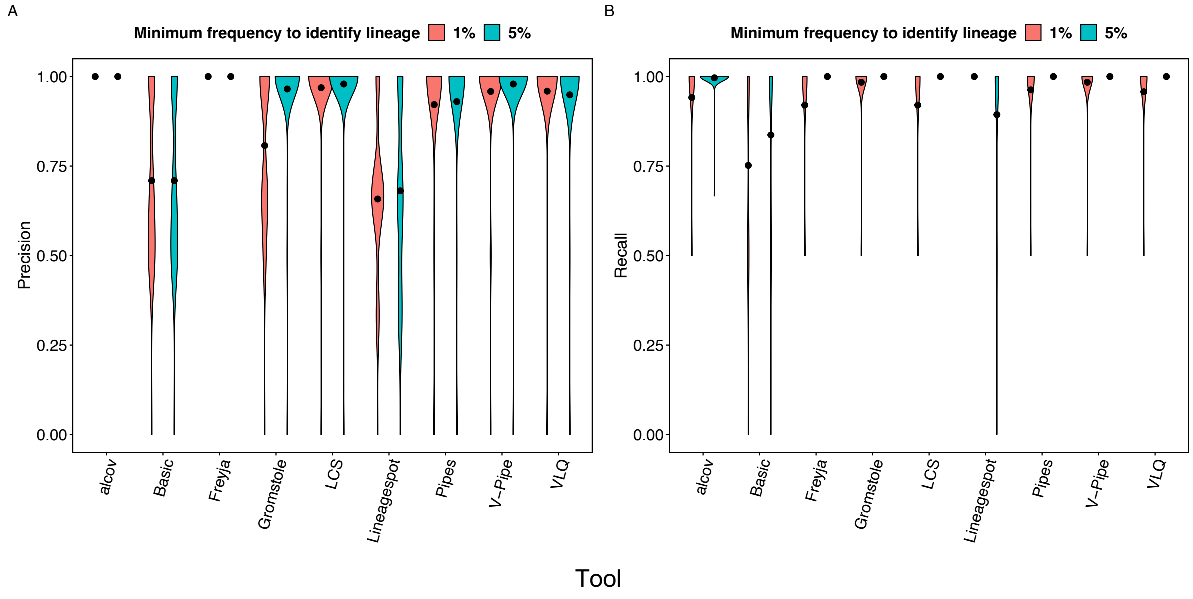

Figure S2. Lineage identification in single-and mixed-lineage samples using alternate minimum frequency cut-offs. Comparisons of A) precision and B) recall with lineage presence defined by ≥ 1% or ≥ 5% cut-offs. Black point shows the mean value.

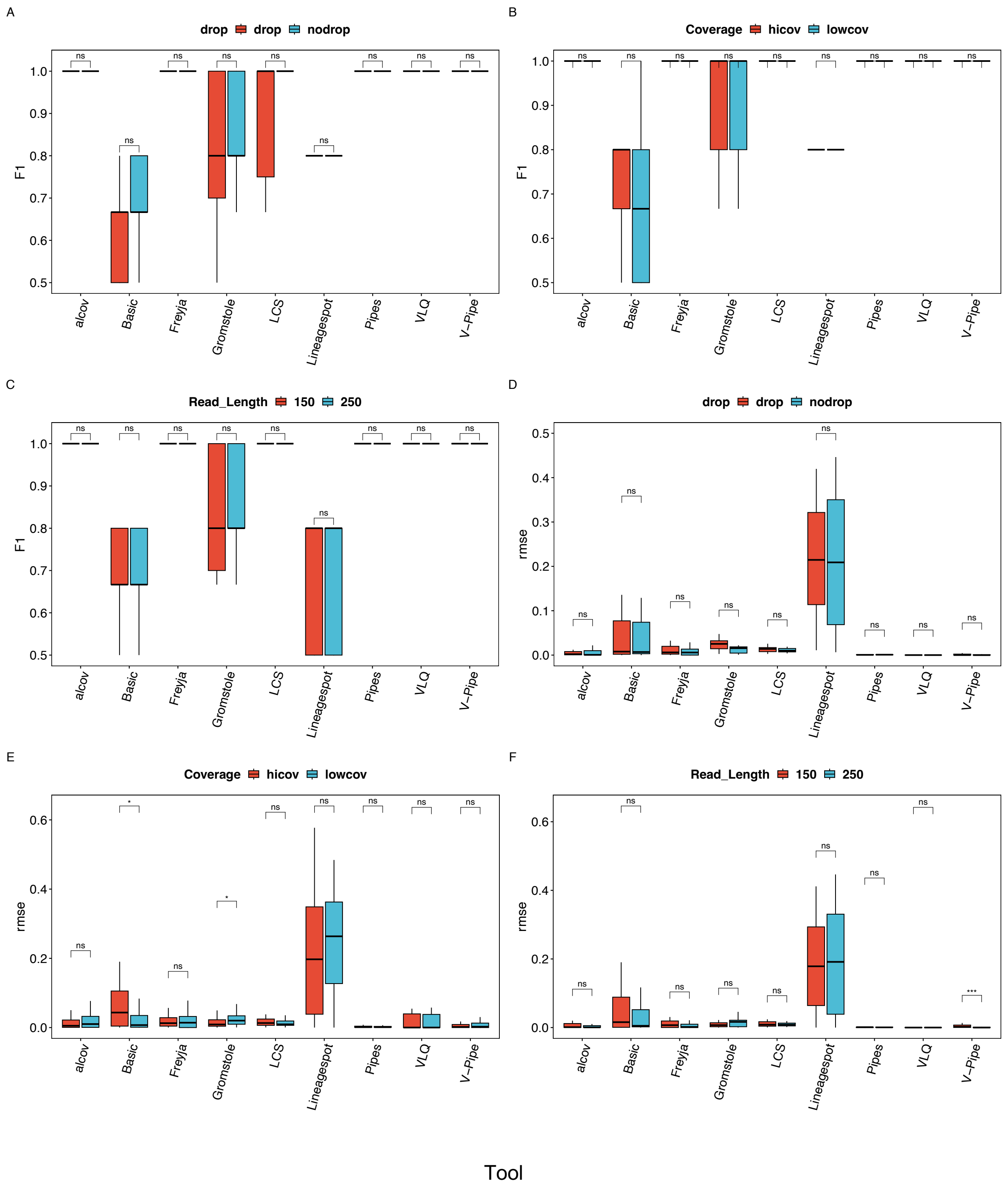

Figure S3. Lineage identification and quantification in single- and mixed-lineage samples. F1-scores and RMSE for (A,D) samples with BA.1 with amplicon dropout (n=13) and matched samples without amplicon drop-out (n=13), (B,E) High-coverage (n=50) and low-coverage samples (n=50), (C,F) 150bp samples (n=24) and their matched 250bp samples (n=24).

Supplementary Table 1. Description of the 100 benchmark samples. *Name* is the category of sample. *Lineages* is the names of lineages present in order of the abundance in the *Lineage ratio*. *Read length* is the length of reads simulated (250bp or 150bp). *Lineage ratio* is the relative abundances of the lineages. *Coverage* is the category of genome coverage simulated with "lowcov" indicating <50x depth and "hicov" indicating 600x.

Name	Lineages	Read length (bp)	Lineage ratio	Coverage
Two lineages	BA.1:Delta	250	75:25	lowcov
Two lineages+Synth	BA.1:Delta:Synth	250	65:25:10	hicov
Two lineages	BA.1:Delta	250	75:25	hicov
Three lineages	BA.1:BA.2:Delta	250	33:33:33	lowcov
Two lineages+Synth	BA.1:Delta:Synth	250	80:10:10	lowcov
Two lineages	BA.1:Delta	150	50:50	lowcov
Three lineages+Synth	BA.1:BA.2:Delta:Synth	250	30:30:30:10	lowcov
Two lineages	BA.1:Delta	250	1:99	lowcov
Two lineages	BA.1:Delta	250	90:10	lowcov
Two lineages	BA.1:Delta	250	90:10	hicov
Three lineages+Synth	BA.1:BA.2:Delta:Synth	250	10:10:70:10	hicov
Two lineages (drop)	BA.1_drop:Delta	250	25:75	hicov
Two lineages	BA.1:Delta	150	10:90	lowcov
Two lineages (drop)	BA.1_drop:Delta	250	90:10	lowcov
Three lineages	BA.1:BA.2:Delta	250	10:80:10	lowcov
Two lineages (drop)	BA.1_drop:Delta	250	1:99	hicov
Single lineage	BA.2	250	100	lowcov
Two lineages	BA.1:Delta	150	75:25	hicov
Two lineages	BA.1:BA.2	250	50:50	hicov
Single lineage	BA.1	150	100	hicov
Two lineages+Synth	BA.1:Delta:Synth	250	1:89:10	hicov
Single lineage	BA.1_drop	250	100	hicov
Single lineage	BA.1_drop	150	100	lowcov
Two lineages (drop)	BA.1_drop:Delta	250	50:50	hicov
Two lineages+Synth	BA.1:Delta:Synth	250	65:25:10	lowcov
Two lineages (drop)	BA.1_drop:Delta	250	50:50	lowcov
Two lineages (drop)	BA.1_drop:Delta	250	75:25	hicov
Two lineages (drop)	BA.1_drop:Delta	250	1:99	lowcov
Two lineages (drop)	BA.1_drop:Delta	250	10:90	hicov
Two lineages+Synth	BA.1:Delta:Synth	250	1:89:10	lowcov

Three lineages+Synth	BA.1:BA.2:Delta:Synth	250	70:10:10:10	hicov
Two lineages	BA.1:Delta	150	25:75	hicov
Two lineages	BA.1:BA.2	250	75:25	hicov
Two lineages	BA.1:Delta	150	99:1	lowcov
Two lineages	BA.1:Delta	150	25:75	lowcov
Two lineages (drop)	BA.1_drop:Delta	250	10:90	lowcov
Two lineages+Synth	BA.1:Delta:Synth	250	45:45:10	lowcov
Two lineages (drop)	BA.1_drop:Delta	250	25:75	lowcov
Two lineages	BA.1:Delta	150	10:90	hicov
Single lineage	BA.1_drop	150	100	hicov
Two lineages	BA.1:Delta	250	25:75	lowcov
Three lineages+Synth	BA.1:BA.2:Delta:Synth	250	10:70:10:10	lowcov
Single lineage	BA.2	150	100	lowcov
Three lineages	BA.1:BA.2:Delta	250	10:10:80	lowcov
Three lineages+Synth	BA.1:BA.2:Delta:Synth	250	70:10:10:10	lowcov
Two lineages	BA.1:Delta	250	99:1	hicov
Two lineages	BA.1:Delta	150	50:50	hicov
Two lineages	BA.1:Delta	250	50:50	lowcov
Two lineages	BA.1:Delta	150	1:99	lowcov
Two lineages	BA.1:Delta	150	75:25	lowcov
Single lineage	Delta	250	100	hicov
Single lineage	Recomb	250	100	lowcov
Two lineages	BA.1:Delta	150	90:10	lowcov
Single lineage	Delta	150	100	lowcov
Two lineages	BA.1:Delta	250	10:90	hicov
Two lineages	BA.1:Delta	150	99:1	hicov
Single lineage	Delta	250	100	lowcov
Two lineages	BA.1:Delta	250	1:99	hicov
Two lineages	BA.1:BA.2	250	50:50	lowcov
Two lineages	BA.1:BA.2	250	25:75	hicov
Two lineages	BA.1:Delta	250	10:90	lowcov

Two lineages+Synth	BA.1:Delta:Synth	250	25:65:10	hicov
Single lineage	BA.1	150	100	lowcov
Two lineages	BA.1:Delta	150	1:99	hicov
Three lineages	BA.1:BA.2:Delta	250	80:10:10	lowcov
Single lineage	Synth	250	100	lowcov
Three lineages	BA.1:BA.2:Delta	250	33:33:33	hicov
Single lineage	Synth	150	100	hicov
Two lineages (drop)	BA.1_drop:Delta	250	75:25	lowcov
Two lineages	BA.1:Delta	150	90:10	hicov
Single lineage	BA.1_drop	250	100	lowcov
Single lineage	Delta	150	100	hicov
Two lineages+Synth	BA.1:Delta:Synth	250	10:80:10	lowcov
Two lineages	BA.1:Delta	250	99:1	lowcov
Single lineage	Synth	250	100	hicov
Single lineage	BA.1	250	100	hicov
Two lineages (drop)	BA.1_drop:Delta	250	99:1	lowcov
Two lineages+Synth	BA.1:Delta:Synth	250	10:80:10	hicov
Three lineages+Synth	BA.1:BA.2:Delta:Synth	250	10:70:10:10	hicov
Two lineages	BA.1:BA.2	250	75:25	lowcov
Two lineages	BA.1:Delta	250	25:75	hicov
Three lineages	BA.1:BA.2:Delta	250	80:10:10	hicov
Single lineage	Recomb	250	100	hicov
Two lineages	BA.1:BA.2	250	25:75	lowcov
Two lineages+Synth	BA.1:Delta:Synth	250	45:45:10	hicov
Two lineages (drop)	BA.1_drop:Delta	250	99:1	hicov
Two lineages+Synth	BA.1:Delta:Synth	250	80:10:10	hicov
Two lineages	BA.1:Delta	250	50:50	hicov
Two lineages+Synth	BA.1:Delta:Synth	250	89:1:10	hicov
Single lineage	BA.2	150	100	hicov
Two lineages+Synth	BA.1:Delta:Synth	250	25:65:10	lowcov
Three lineages	BA.1:BA.2:Delta	250	10:80:10	hicov

Three lineages+Synth	BA.1:BA.2:Delta:Synth	250	10:10:70:10	lowcov
Three lineages+Synth	BA.1:BA.2:Delta:Synth	250	30:30:30:10	hicov
Two lineages+Synth	BA.1:Delta:Synth	250	89:1:10	lowcov
Two lineages (drop)	BA.1_drop:Delta	250	90:10	hicov
Single lineage	BA.1	250	100	lowcov
Single lineage	Synth	150	100	lowcov
Three lineages	BA.1:BA.2:Delta	250	10:10:80	hicov
Single lineage	BA.2	250	100	hicov

Supplementary Table 2. Genomes from GISAID used in building the benchmark. Dataset available on 2022-03-06. GISAID Identifier: EPI_SET_230802sn, DOI: 10.55876/gis8.230802sn, All genome sequences and associated metadata in this dataset are published in GISAID's EpiCoV database. To view the contributors of each individual sequence with details such as accession number, Virus name, Collection date, Originating Lab and Submitting Lab and the list of Authors.

Virus name	Pango designation	EPI_SET ID	Benchmark Category
Australia/QLD2568/2021	BA.2	EPI_ISL_7190366	BA.2
Canada/AB-ABPHL-47244/2021	BA.1.15	EPI_ISL_8595541	BA.1
Canada/BC-BCCDC-188298/2021	AY.25.1	EPI_ISL_4833392	Delta
Canada/BC-BCCDC-326252/2022	BA.1.3	EPI_ISL_9757034	BA.1
Denmark/DCGC-129368/2021	AY.9.2	EPI_ISL_3028262	Delta
Denmark/DCGC-208970/2021	AY.4.10	EPI_ISL_6723534	Delta
Denmark/DCGC-331760/2022	BA.2.3	EPI_ISL_9163335	BA.2
Denmark/DCGC-348305/2022	BA.1.18	EPI_ISL_9505648	BA.1
England/ALDP-17411AC/2021	AY.4	EPI_ISL_2701451	Delta
England/ALDP-335E483/2022	BA.1.16	EPI_ISL_9173628	BA.1
England/BRBR-31D0D35/2022	BA.1.1.13	EPI_ISL_8755967	BA.1
England/LSPA-34C0C65/2022	BA.2.1	EPI_ISL_9586229	BA.2
England/MILK-34B1872/2022	BA.2.5	EPI_ISL_9582210	BA.2
England/RAND-	B.1.617.2	EPI_ISL_1631836	Delta

14DD366/2021			
Finland/11593/2021	AY.46	EPI_ISL_4093267	Delta
Germany/RP-RKI-I-517374/2022	BA.2.5	EPI_ISL_10005779	BA.2
Germany/ST-RKI-I-528761/2022	BA.1.18	EPI_ISL_10021450	BA.1
India/un-LNHD7/2021	BA.1.1	EPI_ISL_7877191	BA.1
Indonesia/KR-NIHRD-WGS-22-00433/2022	BA.2.3	EPI_ISL_9468151	BA.2
Japan/IC-1701/2021	AY.28	EPI_ISL_3392341	Delta
Russia/SPE-RII-6094S/2021	BA.1.1	EPI_ISL_7891529	BA.1
Scotland/QEUH-1797A51/2021	AY.4.2	EPI_ISL_2729292	Delta
Scotland/QEUH-320BC60/2022	BA.1.15.1	EPI_ISL_8822590	BA.1
Scotland/QEUH-35F1D58/2022	BA.1.19	EPI_ISL_9898641	BA.1
Scotland/QEUH-377A098/2022	BA.2.8	EPI_ISL_10337543	BA.2
Scotland/QEUH-3970F93/2022	BA.2.8	EPI_ISL_11035940	BA.2
Singapore/2437/2022	BA.2.4	EPI_ISL_11019970	BA.2
Slovenia/5C-039815-KP/2021	AY.43	EPI_ISL_5105429	Delta
Sweden/5464031987/2021	B.1.617.2	EPI_ISL_2793160	BA.1
Switzerland/BS-UHB-43321728/2022	BA.2.3	EPI_ISL_9755871	BA.2
USA/NJ-CDC-ASC210553977/2022	AY.119.2:BA.1.1	EPI_ISL_10389336	Recombinant
USA/NJ-CDC-ASC210553978/2022	AY.119.2:BA.1.1	EPI_ISL_10389339	Recombinant
USA/PA-CDC-LC0474055/2022	AY.119.2:BA.1.1	EPI_ISL_8981824	Recombinant
USA/PA-CDC-LC0474301/2022	AY.119.2:BA.1.1	EPI_ISL_8981459	Recombinant
